# Supplementary material for: Prognostic value of pre-treatment systemic immune-inflammation index in patients with endometrial cancer
Source: PLoS One. 2021 May 14;16(5):e0248871. doi: 10.1371/journal.pone.0248871 (PMC8121307; doi:10.1371/journal.pone.0248871)
Supplement: S1 Table — (DOCX) [file pone.0248871.s001.docx]

Supplemental Table 1. Correlations between SII for OS and clinicopathological characteristics.

|  |  | SII≤910 (n=300) | SII>910 (n=142) | *p-*value |
| --- | --- | --- | --- | --- |
| Age | ≦59 | 150 | 82 | 0.128 |
|  | >59 | 150 | 60 |  |
| FIGO stage | I | 234 | 77 | <0.001 |
|  | II | 25 | 13 |  |
|  | III | 29 | 27 |  |
|  | IV | 12 | 25 |  |
| Histological subtype | Endometrioid | 246 | 113 | 0.543 |
|  | Non-endometrioid | 54 | 29 |  |
| Histological grade | Grade 1 | 152 | 46 | <0.001 |
|  | Grade 2 | 62 | 31 |  |
|  | Grade 3 ^1^ | 86 | 65 |  |
| LVSI | Positive | 77 | 41 | 0.009 |
|  | Negative | 219 | 92 |  |
|  | Unknown | 4 | 9 |  |
| Peritoneal cytology | Positive | 41 | 27 | 0.004 |
|  | Negative | 255 | 106 |  |
|  | Not performed | 4 | 9 |  |

^1^: Grade 3 includes grade 3 endometrioid type and non-endometrioid type

Abbreviations: SII; systemic immune inflammation index, FIGO; Federation of Gynecology and Obstetrics, LVSI; lymphovascular space invasion, NA; not available, OS; overall survival.
