# Supplementary material for: Prognostic value of pre-treatment systemic immune-inflammation index in patients with endometrial cancer
Source: PLoS One. 2021 May 14;16(5):e0248871. doi: 10.1371/journal.pone.0248871 (PMC8121307; doi:10.1371/journal.pone.0248871)
Supplement: S2 Table — (DOCX) [file pone.0248871.s002.docx]

Supplemental Table 2. Summary of investigations of the role of SII in endometrial cancer patients.

| Author (year) | Patients (number) | Cutoff value of SII | Conclusions |
| --- | --- | --- | --- |
| Holub K, et al (2020)(31). | Stage I-III (n=155) | 1100 | Elevated SII was fund to be an independent unfavorable prognostic factor for both OS and PFS. |
| Mirili C, et al (2020)(32). | Stage I-IV (n=101) | 1035.9 | Elevated SII was fund to be an independent unfavorable prognostic factor for both OS and PFS. |
| Current study | Stage I-IV (n=442) | 931 for PFS  910 for OS | Elevated SII was fund to be an independent unfavorable prognostic factor for both OS and PFS. |

PFS; progression free survival, OS: overall survival, SII; systemic immune inflammatory index
