# Supplementary material for: Prognostic value of pre-treatment systemic immune-inflammation index in patients with endometrial cancer
Source: PLoS One. 2021 May 14;16(5):e0248871. doi: 10.1371/journal.pone.0248871 (PMC8121307; doi:10.1371/journal.pone.0248871)
Supplement: Attachment — Submitted filename: Response to Reviewer.docx [file pone.0248871.s003.docx]

We thank the reviewer for carefully reviewing our manuscript and providing us with useful comments. Our manuscript was revised based on the reviewer’s comments, and a detailed response to each point is presented below.

**Responses to Reviewer #1**

**Comment 1:**

Only question I'd have is the unbalanced arms. The derived systemic immune index is significantly different in for patients in different stages and grades. Would the index still be signifiicant if the arms were balanced with a propensity score matching? 
**Response:**

Thank you for a reviewer’s thoughtful comment. Although SII was found to be an independent predictor for decreased PFS and OS in patients with endometrial cancer in the multivariate analysis, the prognostic significance of SII was not investigated in studies in which arms (SII-high versus SII-low) were balanced. Thus, to validate the results from current study, future studies especially in a prospective randomized or a propensity score matching settings are required. We have described this in lines 267-269 of the revised manuscript.

**Responses to Reviewer #2**

**Comment 1:**

While SII may have been determined to be an independent risk factor for survival, FIGO stage was by for the strongest predictor and this should be acknowledged in the abstract.
**Response:**

As suggested, we have indicated this in the Abstract.

**Comment 2:**

The sample was predominantly women with early disease and highly treatable endometrioid disease - yet "all patients were treated with surgery AND chemotherapy" -were women who only underwent hysterectomy excluded?
**Response:**

Thank you for the reviewer’s comment. We have corrected the wordings (lines 108-111 of the revised manuscript).

**Comment 3:**

The mean BMI was within normal limits - this seems very unusual for endometrial cancer. The discussion should include a description and related context for interpreting the findings in countries where obesity is the major driver of disease risk. At minimum generalizability of the findings needs to be addressed more robustly.
**Response:**

I agree with the reviewer’s comment. It has been generally accepted that high BMI is associated with increased risk of endometrial cancer development. However, BMI of the Japanese women with endometrial cancer is not so high. For example, in a study conducted by Honda T, et al, roughly 70% of Japanese endometrial cancer patients had BMI<25 (Int J Womens Health. 2012;4:207-12.), which is consistent with our study. We think the prognostic significance of SII need to be further investigated in larger studies involving patients with wide range of BMI. We have described this as a limitation of the current study in lines 258-260 of the revised manuscript.

**Comment 4:**

Do you have data on co-morbid conditions such as diabetes? CVD? that may influence survival?
**Response:**

Thank you for a reviewer’s thoughtful comment. We did not include comorbid conditions such as a diabetes or cardiovascular diseases, which may affect patient’s survival, as a prognostic variable in our univariate and multivariate analyses. We have described this as a limitation of the current study in lines 263-265 of the revised manuscript.

**Comment 5:**

How readily assessed are the markers of the SII contstruct? in other words, is it reasonable to apply these over FIGO stage to estimate prognosis -or would it require additional measures be routinely assessed clinically? more clinical context to the application of these measures would be important.

**Response:**

In our multivariate analyses, the prognostic impact of FIGO stage was greater than SII (Table 3). However, whether it is reasonable to apply SII over FIGO stage or vice versa to estimate the prognosis of endometrial cancer patients remains unknown. Thus, we believe that the clinical application of SII in the survival estimation need to be further investigated in this patient population. We have described this as a limitation of the current study in lines 269-273 of the revised manuscript.
