# Supplementary material for: Prognostic value of pre-treatment systemic immune-inflammation index in patients with endometrial cancer
Source: PLoS One. 2021 May 14;16(5):e0248871. doi: 10.1371/journal.pone.0248871 (PMC8121307; doi:10.1371/journal.pone.0248871)
Supplement: Attachment — Submitted filename: Responses to reviewer.docx [file pone.0248871.s004.docx]

**Responses to reviewers**

**Comment 1:** Would be interesting to see the numbers of pts who received chemo, radiation in Table 1.
**Response:** As suggested, we have included the numbers of patients who received chemotherapy or radiotherapy in the revised Table 1.

**Comment 2:** Line 103: Pre-treatment routine blood work is defined as when? Before surgery or before adjuvant therapy?

**Response:** As stated in the title, we have investigated the “**Prognostic value of pre-treatment systemic immune-inflammation index in patients with endometrial cancer**”. However, as the reviewer pointed, the timing of the blood test has not been defined in the “Methods”. We now defined it in the revised manuscript (lines 115-118).

**Comment 3:** Line 108: Clinically staged? Standard for endometrial cancer is surgical staging.

**Response:** Thank you for the reviewer’s thoughtful comment. As pointed, the standard for endometrial cancer is surgical staging. We also do surgical staging for endometrial cancer patients. However, if surgery isn't possible especially in stage IVB patients, patients were clinically staged instead. Clinical staging in such patients are allowed globally. Clinical staging has been based on the results of a physical exam, biopsy, and imaging tests. We have changed the wording regarding the staging (lines 93-94 of the revised manuscript).

**Comment 4:** References

**Responses:** Thank you for a suggestion regarding the recent researches about SII in endometrial cancer. When we submitted our manuscript to PLOS one in Feb 2020, no reports have been published. Thus, in line 75-76 of the original manuscript, we have stated “the prognostic significance of SII in endometrial cancer patients or its relative utility when compared with other inflammatory indexes has never been investigated”. Moreover, in lines 220-221 of the revised manuscript, we stated “To our knowledge, this is the first study that investigated the prognostic significance of SII in endometrial cancer patients”. However, the time has passed, and situation have been changed. As suggested, we have changed the wordings (in lines 197-198 and 206-211 of the revised manuscript), and included recent researches about SII in endometrial cancer in the “References” and a “Supplemental Table 2”.
